# The association between stressful life events and perceived quality of life among women attending infertility treatments: the moderating role of coping strategies and perceived couple’s dyadic adjustment

**DOI:** 10.1186/s12889-019-7925-4

**Published:** 2019-11-21

**Authors:** Maria Clelia Zurlo, Maria Francesca Cattaneo Della Volta, Federica Vallone

**Affiliations:** 1 Department of Political Sciences, University of Naples Federico II, Via L. Rodinò 22, 80138 Naples, Italy; 2 Department of Humanities, University of Naples Federico II, Via Porta di Massa 1, 80133 Naples, Italy

**Keywords:** Infertility, Stressful life events, Quality of life, Coping strategies, Couple’s dyadic adjustment

## Abstract

**Background:**

Research highlighted that Stressful Life Events have high incidence among infertile patients and significant impact on physical and medical parameters related to reproductive functions, but their potential role among factors influencing the infertile patients’ perception of fertility-related Quality of Life (QoL) has not been explored. The present study aims to investigate the associations of Stressful Life Events (Stressful events in the family of origin, In family pre-existing pregnancy difficulties, Health problems in childhood) with perceived fertility-related QoL in women attending infertility treatments, examining the potential moderating role of adopted coping strategies and perceived couple’s dyadic adjustment.

**Methods:**

A questionnaire consisting of Socio-demographics and Infertility-related characteristics, Stress-inducing events in the couples’ lives Questionnaire (FLS), Coping Orientations to Problem Experienced (COPE), Dyadic Adjustment Scale (DAS), and Core and Treatment subscales of Fertility Quality of Life (FertiQoL) was administered to 266 women attending infertility treatments. A descriptive correlational design with cross-sectional comparison was used. **Results** Logistic Regression Analyses after adjusting for socio-demographic and infertility-related characteristics revealed that women who reported Stressful events in the family of origin and In family pre-existing pregnancy difficulties were more likely to report lower levels of perceived Core QoL, while women who reported Health problems in childhood were more likely to report lower levels of perceived Treatment QoL. Couple’s dyadic adjustment and specific coping strategies were significantly associated with perceived Core and Treatment QoL and they also significantly moderated the associations between stressful life events and perceived QoL.

**Conclusions:**

Data provided original evidence on the strong association between stressful life events and perceived fertility-related QoL also highlighting individual and couples’ resources to define counselling interventions with women attending infertility treatments.

## Background

Infertility is clinically described as a disease of the reproductive system defined by the failure to achieve a clinical pregnancy after 12 months or more of regular unprotected sexual intercourse [1], affecting 9–15% of couples worldwide [2]. Research widely recognized that infertility is a distressing experience [3–5] that may significantly affect perceived levels of quality of life (QoL) [6–11] and psychological health [12–15], and implies attempts of re-definition by searching for causes and meanings to re-establish personal control and satisfactory (QoL) [16–18].

QoL is a multidimensional construct defined as the perception of individuals of their position in the context of their culture and values system and in relation to their goals, expectations, standards, and concerns, which comprises psychological, physical, social and environmental perceived functioning and health [19]. In the same perspective, the construct of the fertility quality of life (FertiQoL) has been developed with the aim of specifically address both dimensions of perceived emotional, physical, relational and social QoL (Core FertiQoL), and dimensions of accessibility and perceived quality of services, quality of interactions with the medical staff, physical and psychological consequences of the medical treatments (Treatment FertiQoL) [20–22]. Therefore, research identified several individual factors (i.e., gender [8, 23], age [23–25] and coping strategies [26, 27]), infertility-related factors (i.e., type of diagnosis, type of treatment and duration of infertility [6, 27, 28]), and relational factors (i.e., quality of marital relationship [29–31]), influencing perceived levels of psychological health and QoL.

Research also highlighted that stressful life events have to be considered as factors widely influencing physical and psychological health parameters [32–37] and perceived levels of QoL [38–42]. Therefore, considering infertility research, several studies showed that the presence of stressful events in the family of origin (e.g., divorce, financial problems, deaths, maltreatment), in family pre-existing pregnancy difficulties (e.g., unwanted child, stillborn children, abortion), and health problems in childhood (e.g., injuries, illness, hospitalization) is frequently reported in the biographical background of infertile patients [43–45], revealing a significant impact on relevant physical and medical parameters related to the reproductive functions, such as menstrual cycle regularity [46], semen quality [47], and pregnancy outcome after In Vitro Fertilization (IVF) [48–50].

Nevertheless, according to previous research on couple counseling and therapy with infertile patients [17], the couple’s biographical background may also have a significant impact on the perception and definition of the current infertility experience at individual, relational and social level. Indeed, previous research highlighted that the adaptation process to an aversive event consists of appraisals of both primary control (i.e., appraisal of possibilities to modify the situation to reduce its negative impact) and secondary control (i.e., appraisals of possibilities to modify the appraisal of circumstances to promote adaptation to them). In particular, secondary control appraisal may include different strategies aiming to enhance adjustment, such as interpretative control (i.e., attempts to give meaning on the basis of previous experiences); cognitive control (i.e., attempts to think and redefine the situation), predictive control (i.e., attempts to predict future events to avoid disappointment and pain previously experienced), vicarious control (i.e., attempts to control experience ascribing it to an authoritative other) and retrospective control (i.e., attempts to identify protective factors in a past negative experience with the aim of consider it avoidable in the future) [16]. Consequently, on the basis of the research reported above, previous stressful life events may be hypothesised as influencing the quality and the effectiveness of control strategies adopted by infertile patients and, therefore, their perceived levels of QoL.

### Aims

Although previous studies suggest that stressful life events have a significant impact on essential parameters for the reproductive success, to date, to the best of our knowledge, little is known concerning the associations between the presence of stressful life events and perceived levels of QoL in infertile patients.

Therefore, the present study aims at analysing the potential association of Stressful Life Events (i.e., Stressful events in the family of origin, In family pre-existing pregnancy difficulties, Health problems in childhood) with perceived Core and Treatment QoL in women attending infertility treatments. Furthermore, considering the significant role exerted by individual resources (i.e., coping strategies) [26, 27, 51–55], and by relational resources (i.e., perceived couple’s dyadic adjustment) [3–5, 29, 31] invested in dealing with infertility, the present study aims at testing the potential moderating role of adopted coping strategies and of perceived couple’s dyadic adjustment in the relationship between experienced Stressful Life Events and perceived levels of Fertility-related Core and Treatment QoL.

## Methods

### Study design and participants

The present cross-sectional study was conducted in collaboration with 9 Italian Centres of Assisted Reproduction of Naples, Udine, and Brescia between March 2016 and January 2017. The study was approved by the Ethical Committee of Psychological Research of the University of Naples Federico II and research was performed in accordance with the 1964 Helsinki declaration and its later amendments or comparable ethical standards. Prior to data collection, the Heads of all Departments of the University were informed about the purpose of the study and data collection procedures and their consent was acquired. Chairmen were asked to give the authorization for administering a questionnaire in their centres and, after obtaining their adhesion to the project, women undergoing infertility treatments were asked to participate in the study before their medical appointment. Participants were female members of couples that had been diagnosed with infertility (Male Factor; Female Factor; Combined Male and Female Factor or Unexplained), with all aetiologies of infertility. Overall, 300 women were asked by their physician to participate in the study, and 266 out of 300 women (response rate = 88.67%) completed a questionnaire lasting 20–30 min (one session) in a quiet room setting in the medical centre. One of the authors was present to answer any queries raised by participants.

### Measures

A questionnaire composed of six sections was submitted. All variables and measures investigated are displayed in Table 1.

**Table 1:** Variables considered in the questionnaire and description of measures

Dimensions	Measures	Variables
Socio-demographic Characteristics	Single item Questions (2 items)	Age in years Educational Level (Senior School/ College)
Infertility Related Characteristics	Single Item Questions (3 items)	Type of Diagnosis (Male, Female, Combined, Unexplained) Type of Treatment (IVF, ICSI, IUI) Duration of Infertility in years
Stressful Life Events	*Stress-inducing events in the couples’ lives Questionnaire* (FLS) [43] (3 items)	Stressful events in the family of origin (Absence/Presence) In family pre-existing pregnancy difficulties (Absence/Presence) Health problems in childhood (Absence/Presence)
Coping Strategies	*Coping Orientations to Problem Experienced- New Italian version* (COPE-NVI) [56, 57] (60 items, 5-point scale)	Problem Solving (Cronbach α = 0.83; cut-off = 32.0)
		Positive Attitude (Cronbach α = 0.76; cut-off = 30.9)
		Social Support (Cronbach α = 0.91; cut-off = 27.7)
		Avoidance (Cronbach α = 0.70; cut-off = 23.5)
		Turning to religion (Cronbach α = 0.85; cut-off = 22.7)
Dyadic Adjustment	*Dyadic Adjustment Scale* (DAS) [58, 59] (32 items, 6-point scale)	Total Score (satisfaction, cohesion, consensus, affectional expression) (Cronbach α = 0.93; cut-off = 115.0)
Fertility Quality of Life	*Fertility Quality of Life Questionnaire* (FertiQoL) [20–22] (36 items, 5-point scale)	Core FertiQoL (Cronbach α = 0.92; cut-off = 54.6)
		Treatment FertiQoL (Cronbach α = 0.92; cut-off = 60.4)

### Statistical analysis

Data were analysed using Statistical Package for Social Science (SPSS, Version 21). Firstly, a number of descriptive analyses of socio-demographic and infertility-related characteristics of study participants were conducted. All the study variables were dichotomised. Age and Duration of Infertility were dichotomised referring to the means of our sample. Educational Level (Senior School education/College education), Type of Diagnosis (Male Factor, Female Factor, Combined Factor and Unexplained Factor), Type of Treatment (In vitro fertilization, IVF; Intracytoplasmic sperm injection, ICSI; Intrauterine insemination, IUI), and Stressful Life Events were coded as absence/presence. Coping Strategies, Total Dyadic Adjustment, and FertiQoL subscales scores were dichotomised referring to the cut-off scores of the validation studies reported in Table 1. Therefore, descriptive analyses of frequencies and percentages of the presence of Stressful Life Events, recourse to Coping Strategies, and perception of Total Dyadic Adjustment and Core and Treatment FertiQoL were carried out. Afterward, Logistic Regression Analyses (method: enter, first indicator contrast; entry criterion: *p* < 0.05; removal criterion: *p* > 0.01, and Hosmer and Lemeshow Goodness-of-fit statistic fixed at *p* > 0.05) were separately run to determine main associations of Stressful Life Events, Coping Strategies, and Total Dyadic Adjustment with perceived Core and Treatment FertiQoL. Finally, a further set of Logistic Regression Analyses was carried out to test the potential significant interactions between Stressful Life Events and both adopted Coping Strategies and perceived Total Dyadic Adjustment in predicting perceived levels of Core and Treatment FertiQoL. Relevant Socio-demographic and Infertility-related characteristics (Age, Educational Level, Type of Diagnosis, Type of Treatment and Duration of Infertility) were included in the Logistic Regression Analyses as control variables in order to consider their potential influence on the model parameters. All the different hypotheses have been tested carrying out separated Logistic Regression Analyses.

## Results

Table 2 provides data on Socio-demographic and Infertility-related characteristics. All the 266 women had a diagnosis of primary infertility.

**Table 2:** Socio-demographic and infertility-related characteristics of study participants (*N* = 266)

Characteristics	Value	Range
Age [*Mean* (SD)]	34.2 (3.83)	[22–49]
≤ 34 years [*n* (%)]	146 (54.9%)	
> 34 years [*n* (%)]	120 (45.1%)	
Educational Level [*n* (%)]		
Senior School	171 (64.3%)	
College	95 (35.7%)	
Type of Diagnosis [*n* (%)]		
Male Factor	89 (33.5%)	
Female Factor	74 (27.8%)	
Combined Factor	61 (22.9%)	
Unexplained	42 (15.8%)	
Type of Treatment [*n* (%)]		
IVF	150 (56.4%)	
ICSI	85 (32.0%)	
IUI	31 (11.6%)	
Duration of infertility [*Mean* (SD)]	3.0 (2.38)	[1–17]
≤ 3 years [*n* (%)]	199 (74.8%)	
> 3 years [*n* (%)]	67 (25.2%)	

As regards the presence of Stressful life events, Stressful events in the family of origin (*N* = 130; 48.9%) was the most frequently reported, followed by In Family pre-existing pregnancy difficulties (*N* = 128; 48.1%) and Health problems in childhood (*N* = 102; 38.3%). As regards perceived levels of Core and Treatment QoL, 57 (21.4%) women reported low levels of Core FertiQoL and 80 (30.1%) low levels of Treatment FertiQoL.

As regards the adopted Coping Strategies, the recourse to Social Support was the most frequently reported (*N* = 189; 71.0%), followed by Positive Attitude (*N* = 171; 64.3%), Problem Solving (*N* = 164; 61.6%), Avoidance (*N* = 160; 60.1%), and Turning to Religion (*N* = 89; 33.4%). Finally, as regards perceived couple’s dyadic adjustment, 97 women (36.5%) reported high levels of Total Dyadic Adjustment.

Table 3 displays significant associations emerged from Logistic Regression Analyses. With respect to main effects, findings highlighted that the presence of Stressful events in the family of origin and In family pre-existing pregnancy difficulties was significantly associated with lower perceived levels of Core FertiQoL, while the presence of Health problems in childhood was significantly associated with lower perceived levels of Treatment FertiQoL. Moreover, with respect to main associations of adopted coping strategies with perceived QoL, findings highlighted that high recourse to both active coping strategies (i.e., Positive Attitude and Social Support) and Avoidance/distancing coping strategies was significantly associated with high perceived levels of Core and Treatment FertiQoL, while high recourse to Problem Solving coping strategy was significantly associated with high levels of perceived Treatment FertiQoL. Finally, with respect to main associations of perceived couple’s dyadic adjustment with perceived QoL, data revealed that high perceived levels of Total Dyadic Adjustment were significantly associated with high perceived levels of both Core and Treatment FertiQoL.

**Table 3:** Regressions: stressful life events, coping strategies and couple’s dyadic adjustment against FertiQoL subscales

	Core FertiQoL		Treatment FertiQoL	
	OR	95% CI	OR	95% CI
Stressful events in the family of origin	0.45*	0.32–0.98	0.18	0.52–3.25
In family pre-existing pregnancy difficulties	0.66**	0.37–0.92	0.37	0.24–2.84
Health problems in childhood	0.80	0.59–4.54	0.44*	0.23–0.92
Problem Solving	0.76	0.51–3.43	3.21**	1.98–6.43
Positive Attitude	2.98*	1.76–5.83	3.54*	2.11–8.49
Avoidance	2.57*	1.84–6.33	3.03*	2.11–7.42
Social Support	3.25**	2.24–7.12	2.98*	1.65–6.83
Das Tot	2.56*	1.61–6.89	3.53*	2.80–7.52
Stressful events in the family of origin x Positive Attitude	2.95*	1.52–7.40	2.53	0.83–3.85
Stressful events in the family of origin x Das Tot	2.31*	1.54–7.11	1.87	0.91–3.54
In family pre-existing pregnancy difficulties x Avoidance	0.43*	0.21–0.89	0.39**	0.12–0.81
In family pre-existing pregnancy difficulties x Social Support	3.96**	2.68–9.32	3.24*	2.13–7.32
In family pre-existing pregnancy difficulties x Das Tot	3.15*	1.35–8.31	2.11	0.90–3.24
Health problems in childhood x Problem Solving	3.76 **	2.61–8.21	3.09*	2.34–7.42
Health problems in childhood x Das Tot	2.67	0.89–3.76	3.51**	2.25–8.17

*p < 0.05; **p < 0.01. *Controlled by Age, Educational Level, Type of Diagnosis, Type of Treatment, and Duration of Infertility*

With respect to interaction analyses, data revealed that negative associations between the presence of Stressful events in the family of origin and perceived levels of Core FertiQoL were significantly moderated by the recourse to Positive Attitude coping strategy, and that negative associations between the presence of Health problems in childhood and perceived levels of Treatment FertiQoL were significantly moderated by the recourse to Problem Solving coping strategy. Moreover, negative associations between the presence of In family pre-existing pregnancy difficulties and perceived Core FertiQoL were significantly buffered by the recourse to Social Support coping strategy, and, conversely, significantly increased by the recourse to Avoidance/Distancing coping strategies. Finally, the negative associations between the presence of all types of Stressful Life Events and perceived levels of both Core and Treatment FertiQoL were found significantly moderated by high perceived levels of couple’s dyadic adjustment.

## Discussion

According to previous studies [43–45], data confirmed a significant incidence of all types of Stressful Life Events among the women attending infertility treatments considered, highlighting that 48.9% of women reported Stressful events in the family of origin, 48.1% reported In family pre-existing pregnancy difficulties, and 38.3% Health problems in childhood. Moreover, concerning perceived levels of QoL, data also revealed the impact of infertility experience and medical treatments showing that 21.4% of women reported low levels of Core FertiQoL and 30.1% low levels of Treatment FertiQoL.

Data also supported the hypothesis of a significant association between the presence of Stressful Life Events and low perceived levels of fertility-related Core and Treatment QoL. In particular, it emerged that women reporting both Stressful events in the family of origin and In family pre-existing pregnancy difficulties were more likely to perceive significantly lower levels of Core FertiQoL (i.e., lower levels of perceived relational, emotional, physical and social QoL) when dealing with infertility experience. Moreover, it emerged that women reporting Health problems in childhood were more likely to perceive lower levels of Treatment FertiQoL, inducing to emphasise the role of early experiences of illness and hospitalisation on the infertile patients’ perception of medical treatments. In this perspective, findings suggested that there is a need to increase consideration of the potential impact of Stressful Life Events on the infertility experience and, therefore, the organisation of infertility counseling interventions and therapy before, during and after treatments, should also carefully address the exploration of individual, relational and transgenerational dimensions associated to Stressful Life Events to identify factors influencing individual and couple risk and resources, meanings of wish for a child and motivation for infertility treatment.

Nonetheless, data also highlighted the positive moderating role exerted by specific individual and relational resources, such as the recourse to active coping strategies (i.e., Positive Attitude, Problem Solving, and Social Support) and the perception of a satisfactory couple’s dyadic adjustment. In particular, in infertile women experiencing the presence of stressful events in the family of origin, the recourse to Positive Attitude (i.e., patients’ capability to preserve an active and optimistic attitude) was found having a significant role in enhancing perceived Core QoL. Moreover, in women reporting experiences of health problems in childhood, the adoption of coping strategies centred on Problem Solving (i.e., patients’ capability to actively redefine, understand and face medical aspects of infertility diagnosis and treatments) was found having a positive and significant influence on perceived levels of Treatment QoL. Furthermore, in the presence of more specific in family pre-existing pregnancy difficulties, the recourse to coping strategies centred on Social Support (i.e., patients’ capability to social sharing and emotional disclosure) was found having a significant influence on perceived levels of Core and Treatment QoL. Conversely, the recourse to Avoidance/distancing coping strategies was not adequate to face the psychological impact of the repetition of previous familiar pregnancy difficulties in patients’ current experience, resulting in significantly lower levels of Core and Treatment QoL.

Finally, findings emphasised the role of perceiving a satisfactory and gratifying couple’s relationship, able to fulfil the needs for consensus, cohesion, security, and emotional involvement to counteract the negative impact of all previous stressful and adverse events considered on perceived levels of QoL, by sharing negative feelings, worry and tension related to infertility and medical treatments.

In this perspective, the present study shared the aim of ESHRE guidelines [60] to promote an optimal management of psychosocial care, providing evidence-based information to be applied by professionals from the field of infertility and medically assisted reproduction to enhance tailored and customized interventions by including the evaluation of presence, types, and severity of previous Stressful Life Events as well as of individual and relational resources in terms of coping profiles and couple’s adjustment.

However, despite these findings, some limitations should be mentioned. Firstly, the present research has a cross-sectional design, and therefore no cause - effect relationships between predictors and outcomes investigated can be suggested. Secondly, the measures used were self-report, increasing the risk of social desirability bias. Thirdly, only the moderating role of coping strategies and perceived dyadic adjustment were considered in the present study, while further research could be developed addressing the potential impact of other variables and other personality characteristics (e.g., personality factors, defence mechanisms, attributional styles, control strategies) which may play a role in predicting QoL in infertile patients. Furthermore, future research could also focus on the potential impact of both stressful life events and perceived fertility-related QoL on relevant medical parameters and pregnancy outcomes. Finally, further research should be developed to specifically focus on the impact of Stressful Life Events on fertility-related QoL among male infertile patients, which could give useful information for psychological interventions for both individuals and couples.

## Conclusions

In conclusion, the present study provided new evidence on the associations between the experience of Stressful Life Events and perceived levels of fertility-related QoL in women undergoing infertility treatments, identifying specific risk and protective factors related to their biographical background to be considered for developing targeted counseling interventions to support and enhance optimal adaptation processes to infertility treatments.

## Data Availability

Data and all other materials for this study are kept at the Department of Political Sciences, University of Naples Federico II. The datasets generated and/or analysed during the current study are not publicly available due to the terms of consent to which the participants agreed but available from the corresponding author upon reasonable request.

## Abbreviations

COPE: Coping Orientations to Problem Experienced
DAS: Dyadic Adjustment Scale
FertiQoL: Fertility-related Quality of Life
FLS: Stress-inducing events in the couples’ lives Questionnaire
ICSI: Intracytoplasmic sperm injection
IUI: Intrauterine insemination
IVF: In Vitro Fertilization
QoL: Quality of Life
SPSS: Statistical Package for Social Science
